# Conjunctival and bulbar sporotrichosis as Parinaud’s oculoglandular syndrome acquired by blood inoculation

**DOI:** 10.3205/oc000175

**Published:** 2021-01-28

**Authors:** Adail Orrith Liborio Neto, Tiago Rubim Caetano, Nairacyr Hans Pestana Gervasio, Rachel Camargo Carneiro

**Affiliations:** 1 Federal University of Rio de Janeiro, Campus Macaé (UFRJ), Rio de Janeiro, Brazil

## Abstract

Parinaud’s oculoglandular syndrome (POS) is a clinical condition characterized by granulomatous conjunctivitis associated with homolateral neck pain and anterior preauricular lymphadenopathy. Several reports of this condition occurred and some bacterial etiological agents were identified. However, fungal infections have also been associated, especially sporotrichosis. A 40-year-old female patient complained about a “little ball” in the lower eyelid of the left eye. On ocular examination, visual acuity and fundoscopy were normal. The biomicroscopy revealed a granulomatous lesion in the lower eyelid of the left eye associated with yellowish discharge. The patient returned the next day, reporting worsening of the condition accompanied by low fever, malaise, preauricular and submandibular lymphadenomegaly. The examination showed the evolution of conjunctival edema and various conjunctival granulomas in the lower and upper tarsus of the left eye, a clinical picture compatible with POS. In the investigation of the clinical history, the patient remembered an episode of contact with blood of cats. During the investigation, we discarded differential diagnoses such as tuberculosis, toxoplasmosis, CMV, herpes virus and *Bartonella*. Serology was positive for *Sporothrix*. Treatment with itraconazole 100 mg once daily was started. By the eighth week, the conjunctival granulomas had disappeared, and the medication was discontinued after 90 days of treatment, after about 2 weeks of total remission. According to the literature, there are no cases of primarily ocular manifestation of blood sporotrichosis transmission. However, in the report, the form of transmission of the disease occurred by inoculation by direct contact with the blood of contaminated cats.

## Introduction

Parinaud’s oculoglandular syndrome is a clinical condition characterized by granulomatous conjunctivitis associated with homolateral neck pain and anterior auricular lymphadenopathy [1]. In 1889 Henri Parinaud and Xavier Galezowski described the syndrome, and some etiologic agents were identified, among them: *Mycobacterium tuberculosis*,* Francisella tularensis*,* Treponema pallidum*,* Chlamydia trachomatis*,* Listeria monocytogenes*,* Haemophilus ducreyi* and *B. proteus*. Fungal infections have also been associated with this syndrome, especially sporotrichosis (*Sporothrix schenckii*), blastomycosis (*Blastomyces dermatitidis*), paracoccidioidomycosis (*Paracoccidioides brasiliensis*) and coccidioidomycosis (*Coccidioidesimmitis*) [1], [2]. Viruses, including Epstein-Barr and herpes simplex virus type 1, are rarely associated [3], [4].

Sporotrichosis is a subcutaneous mycosis of worldwide distribution and currently notable in highly endemic areas in Latin America [5]. Caused by the dimorphic fungus *Sporothrix schenckii*, it was first described by Benjamin Schenck in the United States in 1898. The infection is usually acquired by inoculation of the fungus through the skin. The clinical form depends on several factors, such as inoculum size, traumatic inoculation depth, thermal tolerance of the strain, and the host’s immune status. Lesions are usually restricted to the skin, subcutaneous tissue, and adjacent lymphatic vessels [6]. However, reports of bulbar conjunctival sporotrichosis are very rare [7].

## Case description

**Figure 1:**
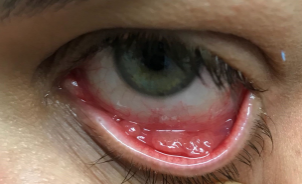
Granulomatous lesion on the lower eyelid of the left eye

A 40-year-old female patient came to the ophthalmology office with a complaint about a “little ball” on the lower eyelid of her left eye two days ago. On eye examination, visual acuity and fundoscopy were normal. Biomicroscopy revealed a granulomatous lesion in the lower eyelid of the left eye associated with yellowish discharge (Figure 1 (Fig. 1)). An association of antibiotics with topical corticosteroids (topical ciprofloxacin and dexamethasone – Cilodex^®^) was prescribed in the left eye, 3x a day, and guided weekly return.

The patient returned the next day, reporting worsening of the condition accompanied by low fever, general malaise, as well as pre-auricular and submandibular lymphadenomegaly. The exam showed the evolution of conjunctival edema and several conjunctival granulomas in the lower and upper tarsus of the left eye, a clinical case compatible with POS. Visual acuity, ocular motility, tonometry and fundoscopy remained unchanged.

In the investigation of the clinical history, where the patient was asked about the possibility of contact with blood of animals, she remembered an episode of contact with blood of cats. She reported that when she arrived to work at the school, she found the classroom completely bloody, including the computer keyboard, after a probable cat fight. The animals had wounds and abrasions with bloody remains, but she denied direct contact with the animals.

In the diagnostic investigation, the following tests were performed: complete blood count, erythrocyte sedimentation rate (ESR), chest X-ray, skin test of purified protein derivative (PPD), acid-resistant staining test (03 samples) and serological tests for toxoplasmosis, CMV, herpes virus, *Bartonella* and *Sporothrix*. Kidney and liver function tests were requested and the infectologist was referred.

**Figure 2:**
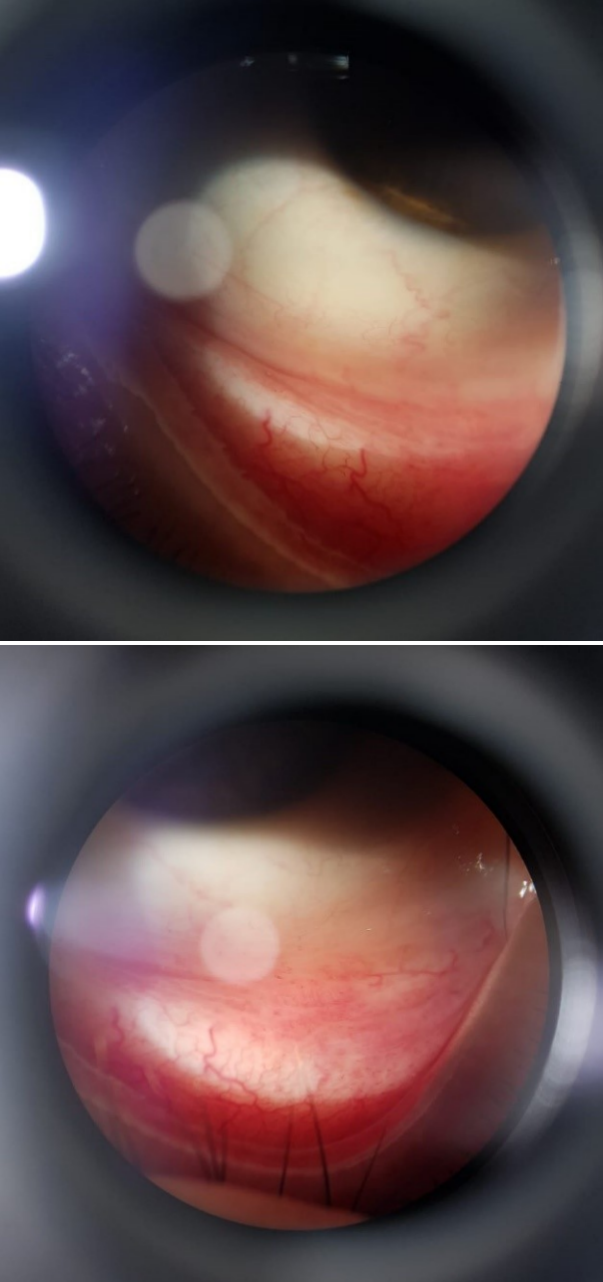
Biomicroscopy showing total lesions on clinical examination

After a week of topical treatment, there was no remission of the lesions. We opted for oral treatment with itraconazole 100 mg once a day and sent the patient to Fiocruz (Fundação Oswaldo Cruz) for diagnostic confirmation of sporotrichosis, maintaining the fortnightly follow-up. In the tenth week of treatment with oral itraconazole, there was complete remission of the lesions, as shown in Figure 2 (Fig. 2), and the 90-day treatment schedule was maintained. The patient remained asymptomatic 6 months after treatment.

## Discussion

Sporotrichosis is a neglected disease without compulsory notification, which makes it difficult to perceive its occurrence and distribution, both in Brazil and worldwide. The disease has been reported in the United States, South America (Brazil, Colombia, Guatemala, Mexico, Peru), Asia (China, India, Japan), and Australia [8]. Other endemically important areas are in China the Northeast of the country, Jillin province, southern Guangdong; in India the north of the country, the sub-Himalayan and Kangra regions; and in Australia the coast of New South Wales and the southeast coast [9]. In Europe, there are few reports, mainly of people who have traveled to endemic areas or by immigration. The countries that report the most cases were France, Italy, and Spain [9]. After the large number of cases reported in France during the beginning of the last century, the number of cases decreased and the disease is rarely reported in Europe [8].

In Peru, an endemic area has been reported in the Andean and Abancay regions. In Mexico, endemic areas can be found in Jalisco and Puebla. Other countries with fewer significant cases are: Colombia, Venezuela, Uruguay, and Guatemala. Brazil has the highest number of cases (Rio de Janeiro, Paraná, Rio Grande do Sul, Minas Gerais, and São Paulo), due to the epidemic related to cats (zoonoses) [9]. In Brazil, Lutz and Splendore 1907, as cited in Orofino-Costa et al. 2017 [10] first described infections in rats and humans, demonstrating the presence of asteroid bodies in tissues, useful for the histopathological diagnosis of this mycosis. Sporotrichosis in Brazil has two reported patterns of transmission: infected dogs and cats, and infected plant matter. Ocular involvement can occur in two ways: by hematogenous dissemination, or by involvement of the ocular attachments through self-inoculation or trauma [8], [9], [10], [11].

The ocular manifestations described in the literature are: granulomatous conjunctivitis, dacryocystitis, granulomatous uveitis, granulomatous retinitis, choroiditis, endophthalmitis, in addition to Parinaud’s oculoglandular syndrome. In the case reported, the form of transmission of the disease was by direct inoculation with the blood of the infected cats, as mentioned by Yamagata et al. [11]. The simultaneous involvement of the ocular mucosa and regional lymph nodes is not uncommon, being one of the causes of the syndrome of Parinaud. Although the cutaneous form is more frequent in Rio de Janeiro and other endemic areas, the ocular presentation has been increasingly diagnosed [10], [11], [12].

According to data in the literature, there are 9 reports of sporotrichosis affecting the eyelids [7], [13], [14], [15], [16], [17], [18], [19], [20]. In the literature, conjunctival injuries were the most observed. Our initial management was based on the presumptive diagnosis of internal hordeolus, and treatment was chosen using the combination of antibiotics and topical corticosteroids. With the appearance of lymphadenomegaly associated with conjunctival granulomatosis lesions, ocular sporotrichosis was suspected.

In the case under study, the patient had no direct contact with the cats. Transmission occurred through contaminated blood, which until now has not been mentioned in the literature. The diagnostic suspicion was confirmed through histopathology, despite the rarity of transmission through this route. In the review by Ramírez Soto [21], it was concluded that the affections are more frequent in the eyelids (n=53, 82%), as described in this report, followed by the lacrimal gland (n=5), conjunctiva (n=4), and eyebrows (n=3).

Although relatively rare, sporotrichosis should be considered in the differential diagnosis of periocular or eyelid lesions that progress despite oral antibiotics, and even in patients without a history of trauma or disseminated infection. The treatment was successful with the use of itraconazole, which is considered the drug of choice due to its efficacy, safety, and dosage convenience, being classified with the scientific evidence level AII [10], [18].

The treatment of lesions affecting the ocular attachments should be treated with itraconazole in the same dose as the cutaneous form: 100–200 mg/day until complete resolution of the lesions (or for an additional 2–4 weeks), in general for a total of 3–6 months [11]. In the review by Bonifaz et al. [9], the authors do not comment on the treatment of ocular sporotrichosis, but only define that the cutaneous-disseminated, disseminated, and pulmonary forms should be treated with amphotericin B, followed by itraconazole and, in cases of severe immunosuppression, the treatment of long-term maintenance with itraconazole.

## Conclusion

According to the literature, there are no cases of transmission of sporotrichosis by blood with primarily ocular manifestation. However, in this report, the form of transmission of the disease occurred through inoculation through direct contact with the blood of the infected cats, confirmed by histopathology and by the success with standard treatment. Although the cutaneous form is more frequent in Rio de Janeiro and other endemic areas, the ocular presentation has increasingly been diagnosed.

## Notes

### Competing interests

The authors declare that they have no competing interests.
